# Gait and Axial Spondyloarthritis: Comparative Gait Analysis Study Using Foot-Worn Inertial Sensors

**DOI:** 10.2196/27087

**Published:** 2021-11-09

**Authors:** Julie Soulard, Jacques Vaillant, Athan Baillet, Philippe Gaudin, Nicolas Vuillerme

**Affiliations:** 1 University Grenoble Alpes AGEIS La Tronche France; 2 Grenoble Alpes University Hospital Grenoble France; 3 University Grenoble Alpes, CNRS, Grenoble Alpes University Hospital, Grenoble INP, TIMC-IMAG UMR5525 Grenoble France; 4 Institut Universitaire de France Paris France; 5 LabCom Telecom4Health, Orange Labs & Univ. Grenoble Alpes, CNRS, Inria, Grenoble INP-UGA Grenoble France

**Keywords:** ankylosing spondylitis, spondyloarthritis, gait, locomotion, pain, mobility, spatiotemporal, digital health, sensors

## Abstract

**Background:**

Axial spondyloarthritis (axSpA) can lead to spinal mobility restrictions associated with restricted lower limb ranges of motion, thoracic kyphosis, spinopelvic ankylosis, or decrease in muscle strength. It is well known that these factors can have consequences on spatiotemporal gait parameters during walking. However, no study has assessed spatiotemporal gait parameters in patients with axSpA. Divergent results have been obtained in the studies assessing spatiotemporal gait parameters in ankylosing spondylitis, a subgroup of axSpA, which could be partly explained by self-reported pain intensity scores at time of assessment. Inertial measurement units (IMUs) are increasingly popular and may facilitate gait assessment in clinical practice.

**Objective:**

This study compared spatiotemporal gait parameters assessed with foot-worn IMUs in patients with axSpA and matched healthy individuals without and with pain intensity score as a covariate.

**Methods:**

A total of 30 patients with axSpA and 30 age- and sex-matched healthy controls performed a 10-m walk test at comfortable speed. Various spatiotemporal gait parameters were computed from foot-worn inertial sensors including gait speed in ms^–1^ (mean walking velocity), cadence in steps/minute (number of steps in a minute), stride length in m (distance between 2 consecutive footprints of the same foot on the ground), swing time in percentage (portion of the cycle during which the foot is in the air), stance time in percentage (portion of the cycle during which part of the foot touches the ground), and double support time in percentage (portion of the cycle where both feet touch the ground).

**Results:**

Age, height, and weight were not significantly different between groups. Self-reported pain intensity was significantly higher in patients with axSpA than healthy controls (*P*<.001). Independent sample *t* tests indicated that patients with axSpA presented lower gait speed (*P*<.001) and cadence (*P*=.004), shorter stride length (*P*<.001) and swing time (*P*<.001), and longer double support time (*P*<.001) and stance time (*P*<.001) than healthy controls. When using pain intensity as a covariate, spatiotemporal gait parameters were still significant with patients with axSpA exhibiting lower gait speed (*P*<.001), shorter stride length (*P*=.001) and swing time (*P*<.001), and longer double support time (*P*<.001) and stance time (*P*<.001) than matched healthy controls. Interestingly, there were no longer statistically significant between-group differences observed for the cadence (*P*=.17).

**Conclusions:**

Gait was significantly altered in patients with axSpA with reduced speed, cadence, stride length, and swing time and increased double support and stance time. Taken together, these changes in spatiotemporal gait parameters could be interpreted as the adoption of a so-called cautious gait pattern in patients with axSpA. Among factors that may influence gait in patients with axSpA, patient self-reported pain intensity could play a role. Finally, IMUs allowed computation of spatiotemporal gait parameters and are usable to assess gait in patients with axSpA in clinical routine.

**Trial Registration:**

ClinicalTrials.gov NCT03761212; https://clinicaltrials.gov/ct2/show/NCT03761212

**International Registered Report Identifier (IRRID):**

RR2-10.1007/s00296-019-04396-4

## Introduction

The generic term spondyloarthritis (SpA) regroups part of chronic inflammatory diseases with common clinical, genetic, and pathophysiological features [1,2]. Diagnosis of SpA is based on the Assessment of Spondyloarthritis International Society (ASAS) criteria [3]. Two groups of SpA are defined: axial SpA (axSpA) with main manifestations being on spinal and sacroiliac joints, and peripheral SpA with main manifestations being arthritis, enthesitis or dactylitis [1,3]. In this study, we will focus on axSpA which is divided into its radiographic (ankylosing spondylitis, AS) and its nonradiographic (nr-axSpA) forms [1,2]. Note that patients with axSpA could represent 0.13% to 1.4% of the world population [4].

Clinical manifestation of axSpA includes chronic inflammatory back pain and morning stiffness [5]. As a consequence of inflammation, structural damage can occur and lead to spinal mobility restrictions [5] associated with restricted lower limb ranges of motion [6], thoracic kyphosis [7], spinopelvic ankylosis [7], decrease in muscle strength [8], and sarcopenia [8]. It is well known that factors such as limited range of motion [9], reduced muscle strength [10], sarcopenia [11], thoracic kyphosis, and spinopelvic alignment [12] can have consequences on spatiotemporal gait parameters during walking.

It is interesting to note that a recent review concluded that no published work has investigated spatiotemporal gait parameters in patients with axSpA [13]. This is not the case for patients with AS, a subgroup of axSpA [3]. A recent review [14] reported that 21 articles assessed gait in AS. Interestingly, only 4 of them (19%) used a healthy control group for comparison of spatiotemporal gait parameters [15-18]. What is more, results of these 4 studies are rather mixed and have reached somewhat inconsistent results and raised unanswered questions [15-18]. Some studies, indeed, reported gait impairment in patients with AS who presented with lower gait speed [18] and lower stride length [16,18] than healthy controls. Other studies reported gait speed [15,17], stride length [15,17], cadence [16,17], swing time, and stance time percentages [18] of patients with AS similar to those of healthy controls. How can we explain these observed differences? It is possible that the relatively small sample size of these studies (from n=10 [17] to n=18 [18] in each group) represented an obstacle to the identification of any significant group differences. Note that this limitation is that of the authors themselves (“However, further study should be performed on a larger sample subjects” [15] and “the sample size was limited” [18]). It is also possible that self-reported pain intensity at the time of assessment played a role in these divergent results. On the one hand, it is recognized that low back pain is one of the main symptoms of axSpA [5] and inflammatory back pain is a central criteria for disease diagnosis [3]. On the other hand, it is also well established that low back pain could significantly affect spatiotemporal gait parameters during walking [19-21]. For instance, previous studies have reported significant differences in spatiotemporal gait parameters between patients with low back pain and healthy matched controls [19,20]. Patients with low back pain presented lower gait speed [19,20] and cadence [20] and shorter stride length [19,20] than healthy matched controls during walking. It is important to mention that self-reported pain intensity at time of evaluation was not reported in all studies on gait and AS. In particular, only studies from Mangone et al [17] and Zhang et al [17] have reported this parameter. Regardless of this, a careful examination and comparative analysis of these two published works [17,18] nevertheless has drawn our attention to more specifically take into consideration the possible impact of pain on spatiotemporal gait parameters during walking. To support this view, let us first consider the work of Mangone et al [17]. Analysis of spatiotemporal gait showed no significant between-group difference for gait speed (AS: 0.94 [SD 0.2] ms^–1^ vs healthy controls: 0.96 [SD 0.2] ms^–1^, *P*=.78) and stride length (AS: 1.09 [SD 0.1] m vs healthy controls: 1.14 [SD 0.2] m, *P*=.40) [17]. Concomitantly, no between-group difference was observed for self-reported pain intensity reported at time of evaluation assessed with the visual analog scale (VAS-AS: 1.0 [SD 1.3] versus healthy controls: 0.7 [SD 1.1]) [17]. Worthy of note also are the very low self-reported pain intensity scores of close to 0. A value of 0 on the VAS is considered as no pain while a value above 3 is considered as moderate pain [22]. In other words, participants of Mangone et al study [17] could thus be considered as pain-free participants.

Unlike the findings of Mangone et al [17], analysis of data from Zhang et al [18] revealed between-group significant difference in spatiotemporal gait. Lower gait speed (AS: 1.15 [SD 0.21] ms^–1^ vs healthy controls: 1.25 [SD 0.09] ms^–1^, *P*=.009) and shorter stride length (stride length/height: AS: 0.70 [SD 0.97] m/m vs healthy controls: 0.76 [SD 0.42] m/m, *P*=.002) were observed in patients with AS (n=18) than in healthy controls (n=18) [18]. Meanwhile, pain intensity scores reported with the VAS in patients with AS only [18] were 3.89 [SD 1.64]. This value is above 3 and hence considered as moderate pain [22]. This self-reported pain intensity score is 3 times higher than that reported by patients with AS involved in the study of Mangone et al [17]. Although self-reported pain intensity was not collected in healthy controls, it is probable that the value for healthy controls would have been close to 0 like in Mangone et al [17] study. Taken together, the two studies have prompted us to consider that inconsistent and inconclusive results on gait in AS could stem from self-reported pain intensity at the time of the evaluation per se. Moreover, inertial measurement units (IMUs) are becoming helpful to assess gait in different populations [23,24]. IMUs allow computation of spatiotemporal gait parameters in clinical practice that are reliable in patients with axSpA. While previous studies demonstrated the advantages of using IMUs in axSpA to assess spinal mobility [25] or level of physical activity [26], no study assessed gait parameters using IMUs in patients with axSpA.

Overall, because of the lack of published works available on gait in patients with axSpA [13] and considering the divergent results obtained in the studies that have assessed spatiotemporal gait parameters in AS [14], which could be partly explained by self-reported pain intensity scores, this study was designed to compare spatiotemporal gait parameters in patients with axSpA and matched healthy individuals without and with pain intensity score as a covariate.

## Methods

### Study Design

The Function, Locomotion, Measurement, Inflammation (FOLOMI) study was approved by local ethics committee (CPP Ile De France 1, RCB: 2017-A03468-45) and registered with ClinicalTrials.gov [NCT03761212] and followed the SPIRIT (Standard Protocol Items: Recommendations for Interventional Trials) checklist. All participants of the study signed informed consent.

### Participants

The sample size of this study was calculated using difference between patients with AS and healthy controls in stride length in the Zebouni et al [16] study with a standard deviation of 0.12, expected difference of 0.14, significance level of 0.05, and power of 80%. The sample size was estimated at 12 in each group using a sample size calculator [27,28]. It was increased to 30 to allow the use of parametric tests.

Data for this cross-sectional study are a subset of individuals recruited in the FOLOMI prospective study that has been described in a previous publication [29]. The first 30 patients with axSpA included in FOLOMI study and 30 age- and sex-matched healthy controls were studied in this work. Inclusion and noninclusion criteria of the FOLOMI study are detailed below for patients with axSpA and for healthy controls in Textboxes 1 and 2.

**Patients with axSpA. axSpA: axial spondyloarthritis**

Inclusion criteria:

- aged 18 to 65 years at time of their first evaluation
- axSpA (based on ASAS criteria [3] or AS (based on modified New York Criteria [30])
- able to walk 180 m without technical help
- with stable treatment for 3 months
- with a public health insurance (French social security)

Exclusion criteria:

- musculoskeletal, cardiorespiratory or neurologic disease that could affect gait
- hip or knee arthroplasty done or planned in the following 18 months
- not able to speak French
- desire of pregnancy in the following 18 months
- adults protected by laws (Article L1121-5)

**Healthy controls.**

Inclusion criteria:

- aged 18 to 65 years at time of evaluation
- able to walk 180 m without technical help
- with a health insurance

Exclusion criteria:

- musculoskeletal, cardiorespiratory or neurologic disease that could affect gait
- hip or knee arthroplasty done
- not able to speak French

### Clinical Characteristics of the Participants

Age, sex, weight, height, self-reported pain intensity at time of evaluation, and pain location were collected for both patients with axSpA and healthy controls by the same observer (JS) [29]. Self-reported pain intensity at time of evaluation score was assessed with the VAS, a horizontal line of 10 cm in length, anchored by word descriptors with no pain on the left side and the worst imaginable pain on the right side [22]. Participants were asked to mark the point corresponding to their current pain. Participants were asked to localized their pain using a pain areas figure [31].

For patients with axSpA only, disease clinical characteristics including treatment, disease duration, and morning stiffness and self-assessment questionnaires including the Bath Ankylosing Spondylitis Functional Index (BASFI) [32] and the Bath Ankylosing Spondylitis Disease Activity Index (BASDAI) [33] were also collected [29].

### Experimental Protocol

Participants performed gait assessments described in a previous publication [29]. In this study, data from the 10-meter walk test (10MWT) in single-task condition only were considered. Participants performed a 10MWT on a 14-meter walkway at comfortable walking speed [34] in single-task condition (3 trials). Gait assessments were performed by the same examiner (JS). Participants wore walking shoes, with 2 inertial measurement units with triaxial accelerometers and gyroscopes (Physilog5, Gait Up), placed above both feet (behind the base of the fifth metatarsal) [35]. The two first and last steps were removed from the analysis [36,37], and at least 16 steps were included in the analysis. For patients with axSpA, regarding the possible consequences of morning stiffness on functional limitations [38], gait assessment was performed at least 2 hours from the end of morning stiffness.

### Spatiotemporal Gait Outcomes

After checking for nonsignificant differences between left and right feet, the following spatiotemporal gait parameters were computed using Gait Analysis Software (version 5.3.0, Gait Up) with the mean of right and left foot values for each trial:

- Speed (ms^-1^): mean walking stride velocity of forward walking
- Cadence (steps/minute): number of steps in a minute
- Stride length (m): distance between two consecutive footprints on the ground, from the heel of a foot to the heel of the same foot, one cycle after
- Swing time (%): portion of the cycle during which the foot is in the air and does not touch the ground
- Stance time (%): portion of the cycle during which part of the foot touches the ground
- Double support time (%): portion of the cycle where both feet touch the ground

The mean between trial 2 and 3 was calculated for each spatiotemporal gait parameter as it has recently been shown to be the more reliable to assess spatiotemporal gait parameters when performing a 10MWT at comfortable speed [35].

### Data Analysis

Data analysis were performed using SPSS (version 20, IBM Corp) and Excel (Microsoft Corp). Independent samples *t* tests were used to compare patients with axSpA and healthy controls in terms of age, gender, height, weight, self-reported pain intensity scores, and spatiotemporal gait parameters.

In the interest of further discerning differences that could exist as a function of group versus changes in self-reported pain intensity scores, the spatiotemporal gait parameters were further analyzed between groups using 1-way analyses of covariance (ANCOVAs) with the addition of pain intensity score as a covariate. Statistical threshold for all analyses was set at *P*=.05. Effect size (Cohen *d* and partial *η*²) and 95% confidence intervals were also calculated.

## Results

### Demographic and Clinical Assessments

Demographic and clinical assessments for patients with axSpA and healthy controls are shown in Table 1. When comparing patients with axSpA and healthy controls, there were no significant differences for age, height, or weight, but patients with axSpA had higher self-reported pain intensity (*P*<.001; Table 1). In healthy controls, pain was located at the low back (1/30, 3%), knees (1/30, 3%), or shoulders (1/30, 3%). In patients with axSpA, pain was located at the low back (17/30, 57%), bottom or sacroiliac joints (12/30, 40%), thoracic back (11/30, 37%), cervical back (14/30, 47%), sternum or ribs (1/30, 3%), hips (6/30, 20%), knees (9/30, 30%), ankle or feet (3/30, 10%), shoulders (6/30, 20%), elbows (4/30, 13%), or hands (5/30, 17%).

Table 1 also presents pharmacological treatments and disease characteristics for patients with axSpA. Most patients with axSpA included in this study had anti-TNF treatment (21/30, 70%), low disease activity with BASDAI <4 (BASDAI: 3.04 [SD 1.90]), and low impact of axSpA on physical function (BASFI: 2.86 [SD 2.04]).

**Table 1:** Patients with axSpA and healthy controls clinical characteristics (n=60).

Clinical characteristics			Healthy controls (n=30)	Patients with axSpA^a^ (n=30)	Independent *t* test				
					*t*		*P* value		95% CI
**Demographics**									
	Age (years), mean (SD)	45.70 (10.60)		45.37 (10.54)	0.121	.90		–5.13 to 5.79	
	Gender (male), n (%)	20 (67)		20 (67)	—^b^	—		—	
	Weight (kg), mean (SD)	70.25 (10.27)		74.15 (12.94)	–1.294	.20		–9.94 to 2.13	
	Height (cm), mean (SD)	174.47 (7.48)		170.77 (7.82)	1.873	.07		–0.25 to 7.65	
	Self-reported pain intensity scores at time of evaluation, mean (SD)	0.20 (0.66)		3.12 (2.38)	–6.463	<.001		–3.82 to –2.02	
**Pharmacological treatment, n (%)**									
	Anti-TNF^c^	0 (0)		21 (70)	—	—		—	
	Anti-IL-17A^d^	0 (0)		2 (7)	—	—		—	
	DMARDs^e^	0 (0)		3 (10)	—	—		—	
	NSAIDs^f^	0 (0)		7 (23)	—	—		—	
	Pain relief	0 (0)		7 (23)	—	—		—	
	No treatment	30 (100)		3 (10)	—	—		—	
**Disease, mean (SD)**									
	Disease duration from diagnosis (years)	—		11.77 (10.11)	—	—		—	
	BASDAI^g^	—		3.04 (1.90)	—	—		—	
	BASFI^h^	—		2.86 (2.04)	—	—		—	
	Morning stiffness duration (min)	—		28.17 (33.71)	—	—		—	

^a^axSpA: axial spondyloarthritis.

^b^Not applicable.

^c^Anti-TNF: antitumor necrosis factor.

^d^Anti-IL-17A: anti-interleukine-17A.

^e^DMARD: disease modifying antirheumatic drug.

^f^NSAID: nonsteroidal anti-inflammatory agent.

^g^BASDAI: Bath Ankylosing Spondylitis Disease Activity Index.

^h^BASFI: Bath Ankylosing Spondylitis Functional Index.

### Spatiotemporal Gait Parameters

Spatiotemporal gait parameters for patients with axSpA and healthy controls are shown in Table 2. Independent sample *t* tests without covariate indicated that patients with axSpA presented lower gait speed (*P*<.001) and cadence (*P*=.004), shorter stride length (*P*<.001) and swing time (*P*<.001), and longer double support time *P*<.001) and stance time (*P*<.001) than matched healthy controls (Table 2).

ANCOVA comparisons of spatiotemporal gait parameters between groups revealed that a significant effect of group was found (*F*: 3.434, *P*=.004, partial *η*²: 0.320). Results for each spatiotemporal gait parameter can be found in Table 2. When using self-reported pain intensity score as a covariate, spatiotemporal gait parameters were still significant with patients with axSpA exhibiting lower gait speed (*P*<.001), shorter stride length (*P*=.001) and swing time (*P*<.001), and longer double support time (*P*<.001) and stance time (*P*<.001) than matched healthy controls except for cadence which was not significant (*P*=.17; Table 2).

**Table 2:** Spatiotemporal gait parameters obtained in patients with axSpA and healthy controls in single-task condition with t test and ANCOVA results when taking self-reported pain intensity as a covariate.

Spatiotemporal gait parameters	Healthy controls (n=30), mean (SD)	Patients with axSpA^a^ (n=30), mean (SD)	Independent *t* test					ANCOVA^b^		
			*t*	*P* value	Cohen *d*	95% CI	*F*		*P* value	Partial *η²*
Speed (ms^-1^)	1.50 (0.16)	1.27 (0.17)	5.528	<.001	1.17	0.15 to 0.32	15.268		<.001	0.211
Cadence (steps/min)	113.89 (6.35)	108.41 (7.85)	2.97	.004	0.72	1.79 to 9.17	1.922		.17	0.033
Stride length (m)	1.56 (0.14)	1.38 (0.15)	4.679	<.001	1.04	0.10 to 0.25	13.508		.001	0.192
Double support time (%)	19.43 (3.42)	22.99 (2.50)	–4.609	<.001	–1.03	–5.11 to –2.01	13.948		<.001	0.197
Swing time (%)	39.84 (1.77)	38.20 (1.19)	4.201	<.001	0.96	0.86 to 2.41	14.011		<.001	0.197
Stance time (%)	60.16 (1.77)	61.80 (1.19)	–4.201	<.001	–0.96	–2.41 to –0.86	14.011		<.001	0.197

^a^axSpA: axial spondyloarthritis.

^b^ANCOVA: one-way analysis of covariance using pain as covariate.

## Discussion

### Principal Findings

Only a few studies have assessed gait in the broader spectra of axSpA [13,26,39]. What is more, these studies have used clinical measurements of gait (ie, 6-min walk test [26] or 6-meter maximum velocity test [39]) without a healthy control group for comparison. Inconsistent results were found in patients with AS regarding spatiotemporal gait parameters [15-18], which may be explained by the rather small sample sizes of these studies and by self-reported pain intensity scores reported by the patients at the time of the evaluation.

This study was hence specifically designed to evaluate and compare spatiotemporal gait in 30 patients with axSpA and 30 matched healthy controls without and with pain intensity score as a covariate.

We found that patients with axSpA walked with reduced speed, cadence, stride length, and swing time and increased double support and stance time and that pain could per se partly explain this gait behavior. These results are in line with those recently reported by Zhang et al [18]. However, it should be noted that we further broaden the range of patients by including patients with axSpA, including AS and nr-axSpA, while Zhang et al [18] assessed gait in patients with AS and with hip involvement only. To our knowledge, this is the first study comparing spatiotemporal gait parameters in the broad range of patients with axSpA and matched healthy individuals [13]. Zhang et al [18] used a 3D motion-capture system, which is hardly accessible to clinical routine, while we used IMUs positioned on the feet, allowing computation of spatiotemporal gait parameters in clinical practice or in an ecological environment [23,40]. Finally, contrary to the Zhang et al [18] study, we included pain as a covariate to examine whether and to what extent self-reported pain intensity score could explain the gait differences observed between patients with axSpA and healthy controls.

Our results first showed a significant decrease of gait speed (control: 1.50 [SD 0.16] vs axSpA: 1.27 [SD 0.17] m/s, Δ=–16.6%, *P*<.001) of patients with axSpA as compared to matched healthy controls. This statistically significant difference is accompanied by a Cohen *d* effect size of 1.17, hence suggesting that the between groups difference for the gait speed is large (*d*>0.8) [41]. In the absence of published work on gait in patients with axSpA [13] and although the included population was broader (axSpA vs AS), we were inclined to compare our results with those obtained in patients with AS. With this in mind, our result is in line with that reported in patients with AS by Zhang et al [18], who compared 18 patients with AS to 18 healthy matched controls (control: 1.25 [SD 0.09] vs AS: 1.15 [SD 0.21] m/s, Δ=–8.3%, *P*=.009). Gait speed of patients and healthy controls measured in this study was slightly higher than that reported in Zhang et al [18] (this study axSpA: 1.27 [SD 0.17] vs Zhang et al [18] AS: 1.15 [SD 0.21] m/s, Δ=–9.9%; this study control: 1.50 [SD 0.16] vs Zhang et al [18] control: 1.25 [SD 0.09] m/s, Δ=–16.6%). If gait were assessed along 10 meters in both studies, Zhang et al [18] included gait initiation, steady-state walking, and gait termination in the analysis. In our study, the acceleration and deceleration phases achieved during gait initiation and termination were not included. We used a 14-meter walkway [42-44] and removed the two first and the two last steps of the trials [36,37], as previously proposed in other studies that have assessed spatiotemporal gait parameters during walking [45-47]. When compared to other studies on AS, our result on gait speed does not corroborate those of Del Din et al [15] (12 AS vs 12 controls, control: 1.12 [SD 0.25] vs AS: 1.05 [SD 0.23] m/s, Δ=–6.45%, *P*=.33) and Mangone et al [17] (17 AS vs 10 controls, control: 0.96 [SD 0.2] vs AS: 0.94 [SD 0.2] m/s, Δ=–2.1%, *P*=.78), who did not report any significant between-group differences for the gait speed.

Our results further showed a significantly shorter stride length in patients with axSpA than in matched healthy controls (control: 1.56 [SD 0.14] vs axSpA: 1.38 [SD 0.15] m, Δ=–12.2%, *P*<.001) with a large Cohen *d* effect size of 1.04. This result is in agreement with the decrease in stride length of patients with AS observed in two previous studies by Zebouni et al [16] (12 AS vs 11 controls, control: 0.72 [SD 0.13] vs AS: 0.58 [SD 0.11] m, Δ=–21.5% , *P*<.05) and Zhang et al [18] (stride length/height: control: 0.76 [SD 0.42], AS: 0.70 [SD 0.97], Δ=–8.2%, *P*=.002). However, our result is not in line with two other studies on AS by Del Din et al [15] and Mangone et al [17], who did not report any significant differences in stride length between AS and controls (control: 1.29 [SD 0.30] vs AS: 0.98 [SD 0.58] m, Δ=–27.3%, *P*=.27 [15]; control: 1.14 [SD 0.2] vs AS: 1.09 [SD 0.1] m, Δ=–4.48%, *P*=.40 [17]).

Our results further revealed a significant reduction of cadence in patients with axSpA as compared to matched healthy controls (control: 113.89 [SD 6.35] vs axSpA: 108.41 [SD 7.85] steps/min, Δ=–4.9%, *P*=.004) with a medium effect size (Cohen *d*: 0.72). This result does not support the previous findings of Zhang et al [18], Zebouni et al [16], or Mangone et al [17], as no significant difference of cadence between patients with AS and healthy controls was observed (control: 0.94 [SD 0.04] vs AS: 0.95 [SD 0.09] /s, Δ=1.06%, *P*=.601 [18]; control: 103.2 [SD 6.6] vs AS: 102.6 [SD 9] steps/min, Δ=–0.58%, *P*=nonsignificant [16]; control: 101.4 [SD 8.7] vs AS: 102.4 [SD 13.3] steps/min, Δ=0.98%, *P*=.65 [17]).

In addition to these three routinely used spatiotemporal gait parameters, we further computed temporal distribution of gait cycle phases using swing time, stance time, and double support time percentages. The distribution of swing and stance period are temporal indicators of gait pattern [48] and often used as objectives in gait rehabilitation [49]. Indeed, the percentage times spent on swing and stance phases are determined by various factors including balance [50] and push-off force generation responsible for step asymmetry in chronic hemiparesis [51] and are associated with gait speed [52]. Only one study on patients with AS assessed these two temporal parameters [18]. Our results showed shorter swing time percentages (control: 39.84% [SD 1.77%] vs axSpA: 38.20% [SD 1.19%] of gait cycle, Δ=–4.2%, *P*<.001, Cohen *d*: 0.96) and longer stance time percentages (control: 60.16% [SD 1.77%] vs axSpA: 61.8% [SD 1.19%] of gait cycle, Δ=2.69%; *P*<.001, Cohen *d*: –0.96) in patients with axSpa than matched healthy controls. Once again, our results are not in agreement with the existing literature as no significant difference with healthy controls of swing period was found by Zhang et al [18] (right: control: 38.61% [SD 1.55%] vs AS: 38.29% [SD 2.62%] of gait cycle, Δ=–0.83%, *P*=.64; left: control: 38.49% [SD 1.66%] vs AS: 38.12% [SD 3.95%] of gait cycle, Δ=–0.97%, *P*=.57).

Our results further showed longer double support time percentages in patients with axSpa than matched healthy controls (control: 19.43% [SD 3.42%] vs axSpA: 22.99% [SD 2.5%] of gait cycle, Δ=16.8%, *P*<.001, Cohen *d*:–1.03). Note that the Cohen *d* effect size for double support time can be considered as large (>0.8). Interestingly, double support time percentage values obtained in this study cannot be compared to other studies as this parameter has never been assessed in AS [14].

To conclude, both the results of this study and those published elsewhere revealed a remarkable lack of consensus in the academic literature on gait and AS, although the low number of published studies and various methodologies make comparisons rather difficult. What explanation could we have for these differences?

Note that the demographic and clinical characteristics of patients and healthy controls (age, weight, and height) and disease duration of patients with axSpA involved in this study (age: 45 years, disease duration: 11.77 years) were comparable to those reported in previous studies (age between 38 and 49.4 years [15-18]; disease duration between 9.3 and 15 years [15-18]) and hence may not account for the observed divergent results.

We further assessed if divergent results previously reported on gait in AS [14] could be partly explained by self-reported pain intensity score at the time of the evaluation per se. The second statistical analysis presented in this study showed that when adjusting for self-reported pain intensity, patients with axSpA still presented lower gait speed, shorter stride length and swing time, and longer double support time. Interestingly, our results also revealed that there were no longer statistically significant between-group differences observed for the cadence. Taken together, these results suggest that differences between groups on cadence observed in this study could thus stem from self-reported pain intensity at the time of the evaluation per se and could explain why previous studies in AS did not find significant differences in cadence [16,17] and reported low pain intensity in patients [17]. In a complementary way, results also suggest that differences between groups on the other spatiotemporal gait parameters observed in this study could not stem from self-reported pain intensity at the time of the evaluation per se. In other words, conclusions should be made with caution with respect to the influence of pain. Whether or not self-reported pain intensity per se could play a role in gait impairment observed in patients with axSpA still remains an open, unresolved question.

**Figure 1:**
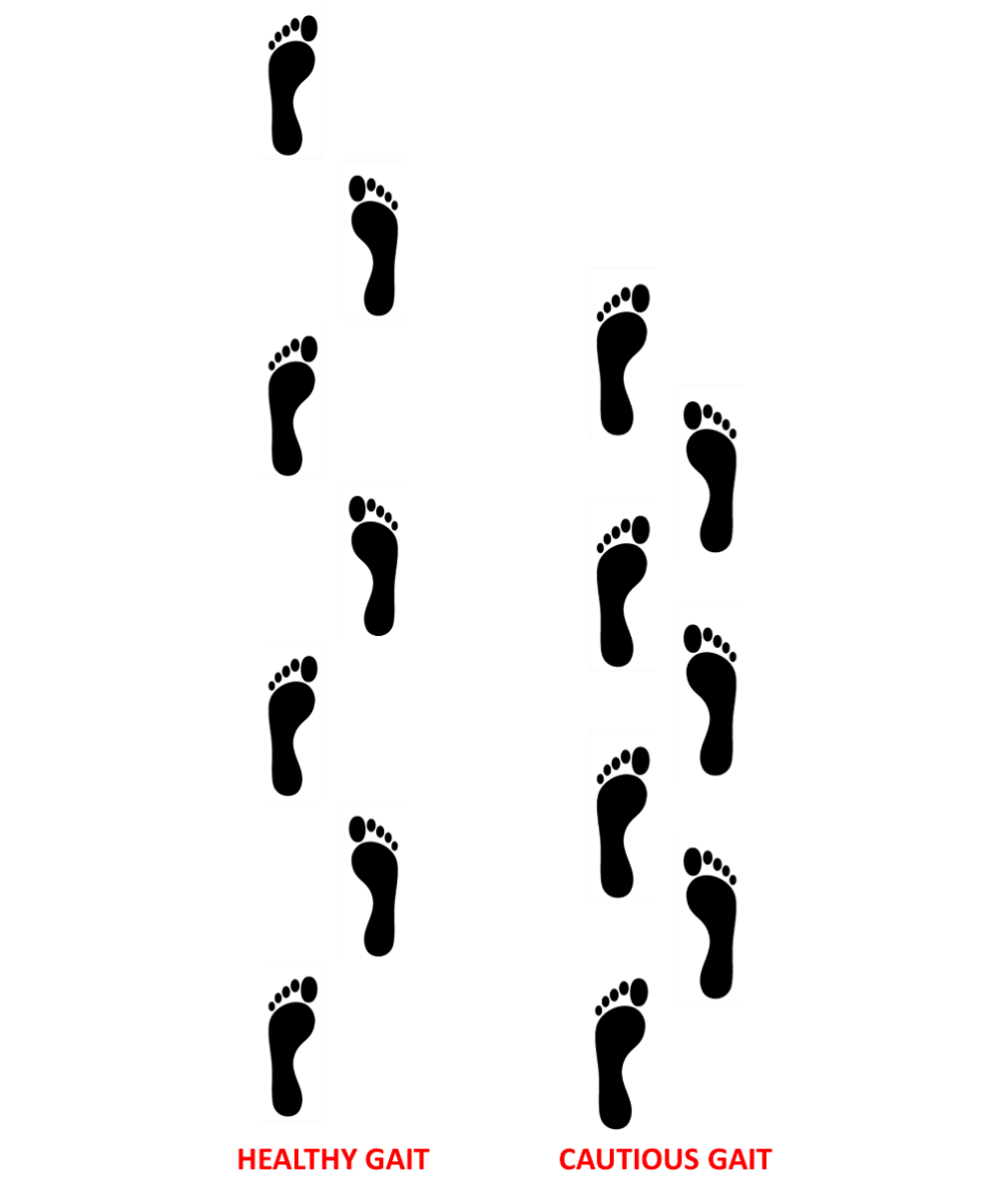
Illustration of a healthy gait and a cautious gait pattern characterized by reduced gait speed and cadence, shortened stride length, and increased double support time.

To synthesize the findings, patients with axSpA presented lower gait speed and cadence, shorter stride length and swing time, and longer double support time and stance time than matched healthy controls during walking. Taken together and looked into as a whole, these changes in spatiotemporal gait parameters could be interpreted as the adoption of a more conservative or less destabilizing gait in patients with axSpA (Figure 1). These results represent the characteristically so-called cautious gait pattern commonly observed in older persons [53] but also in individuals with gait disorders (eg, patients with cerebellar ataxia [54], with sensory ataxia [54], adults with obesity [55-57], and with low back pain [19-21]). This typical characteristic of cautious gait has already been observed in patients with AS [15,16]. However, these studies found that stride length was significantly shortened [16] or found only “a trend towards reduction” in gait speed or stride length [15] in patients with AS as compared to controls. Overall, it has been emphasized that individuals compensate for their balance disorders and/or gait by being more cautious during walking. Hence, adopting a more conservative gait pattern, characterized in particular by a slow gait speed, shortened stride/step length, reduced cadence, and an increased time spent in double limb support could be viewed as an adaptation to ensure or increase stability and maintain a safe gait [53,58].

It is important to note that the differences in spatiotemporal gait parameters obtained between patients with axSpA and healthy controls were outside the standard error of measurement and minimal detectable change (MDC), the minimum value for which a difference can be considered as real [59]. MDC adapted to our group (〖MDC〗_group=〖MDC〗_(individual )÷√n [59,60]) was 0.01 for speed and stride length, between 0.74 and 0.92 for cadence, between 0.24 and 0.53 for double support, and between 0.25 and 0.28 for swing time and stance time [35]. Accordingly, the significant between-group differences observed for these 6 spatiotemporal gait parameters cannot be considered as a measurement error. All in all, our findings showed that patients with axSpA adopted a cautious gait pattern in a similar fashion as the previously mentioned populations [20,53-57].

Taken together, the results of this study are promising for clinical application of gait analysis. We demonstrated that assessing gait in patients with AS using foot-worn inertial sensors is feasible in clinical settings. Spatiotemporal gait parameters (such as stride length or cadence) are the most used parameters in clinical gait analysis and are easy to understand by both clinicians [23] and patients. IMUs, by allowing rapid and easy-to-perform computation of spatiotemporal gait parameters at a low cost and without limitation of the testing environment, are gaining interests for clinicians [23,61]. The 10MWT used in this study is also routinely used by physiotherapists or medical doctors to evaluate gait in clinical and rehabilitation settings. In addition to the time taken to complete this test [62,63], foot-worn inertial sensors enabled the quantitative gait patterns analysis of patients with axSpa with the computation of spatiotemporal gait parameters that were presented in an intuitive and comprehensible manner. We believe that integrating quantitative gait analysis with wearable IMU systems for clinical assessments could be advantageous for clinicians to better understand movement-related disorders for better functional diagnosis, guidance of treatment planning, monitoring of disease progress, and tracking of recovery [64]. In the near future, we can expect that mobile phone–based gait assessment apps will be used to monitor gait in daily life [65] and permit clinicians to remotely monitor patients’ conditions [66,67].

### Limitations

Some limitations of the study should be acknowledged. First, patients included in the study were aged between 18 and 65 years with a pathology evolving with age and an increase of stiffness and limitations. Assessments of older patients could be interesting to capture gait alterations associated with disease evolution. Second, although self-reported pain intensity measured at time of assessment was significantly higher in patients with axSpA than healthy controls, levels of pain were quite low (3.12 [SD 2.38]). Patients included in this study were stable (ie, with stable treatment for at least 3 months at time of inclusion) and may not represent the whole population of patients with axSpA [68]. Further studies are thus necessary to explore gait in the broad disease of axSpA. Patient-reported pain intensity is commonly measured with the single VAS. However, VAS alone may not capture all features of pain [69,70] and may be not sufficient to assess pain in patients with axSpA [71]. Finally, additional research is required to determine whether factors other than pain may influence gait in patients with axSpA.

### Conclusions

To our knowledge, this is the first study comparing spatiotemporal gait parameters in a broad range of patients with axSpA and matched healthy individuals. Our results provide a comprehensive overview of the alterations of gait in patients with axSpA with reduced speed, cadence, stride length, and swing time and increased double support and stance. When all these changes in spatiotemporal gait parameters are taken together and looked into as a whole, it is possible to consider that patients with axSpA adopt a so-called cautious gait pattern. It is the first study to include pain intensity as a covariate to explain spatiotemporal gait parameters in patients with AS or axSpA. Although not a definitive finding, our results suggest that among factors that may influence gait in patients with axSpA, patient self-reported pain intensity could play a role and hence should be addressed when assessing gait in this population.

## Abbreviations

10MWT: 10-meter walk test
AGEIS: Autonomy, Gerontology, eHealth, Imaging, and Stroke
ANCOVA: one-way analysis of covariance
AS: ankylosing spondylitis
ASAS: Assessment of Spondyloarthritis International Society
axSpA: axial spondyloarthritis
BASDAI: Bath Ankylosing Spondylitis Disease Activity Index
BASFI: Bath Ankylosing Spondylitis Functional Index
FOLOMI study: Function, Locomotion, Measurement, Inflammation study
IMU: inertial measurement unit
nr-axSpA: nonradiographic axial spondyloarthritis
SpA: spondyloarthritis
SPIRIT: Standard Protocol Items: Recommendations for Interventional Trials
VAS: visual analog scale
